# Baicalin modulates microRNA expression in UVB irradiated mouse skin

**DOI:** 10.1016/S1674-8301(12)60022-0

**Published:** 2012-03

**Authors:** Yang Xu, Bingrong Zhou, Di Wu, Zhiqiang Yin, Dan Luo

**Affiliations:** Department of Dermatology, the First Affiliated Hospital of Nanjing Medical University, Nanjing, Jiangsu 210029, China.

**Keywords:** microRNA, microarray, ultraviolet radiation B, photodamage, baicalin

## Abstract

This study aimed to evaluate the effects of baicalin on ultraviolet radiation B (UVB)-mediated microRNA (miRNA) expression in mouse skin. We determined miRNA expression profiles in UVB irradiated mice, baicalin treated irradiated mice, and untreated mice, and conducted TargetScan and Gene Ontology analyses to predict miRNA targets. Three miRNAs (mmu-miR-125a-5p, mmu-miR-146a, and mmu-miR-141) were downregulated and another three (mmu-miR-188-5p, mmu-miR-223 and mmu-miR-22) were upregulated in UVB irradiated mice compared with untreated mice. Additionally, these miRNAs were predicted to be related to photocarcinogenesis, hypomethylation and apoptosis. Three miRNAs (mmu-miR-378, mmu-miR-199a-3p and mmu-miR-181b) were downregulated and one (mmu-miR-23a) was upregulated in baicalin treated mice compared with UVB irradiated mice, and they were predicted to be related to DNA repair signaling pathway. These deregulated miRNAs are potentially involved in the pathogenesis of photodamage, and may aid treatment and prevention of UVB-induced dermatoses.

## INTRODUCTION

Ultraviolet (UV) radiation, particularly its ultraviolet radiation B (UVB) component (290-320 nm), is the major cause of skin cancer. UV radiation is also known to elicit various other adverse effects such as erythema, sunburn, inflammation, hyperplasia, hyperpigmentation, immunosuppression, premature skin aging, and photocarcinogenesis[1],[2]. These cellular events are mediated by gene activation, suppression[3] or DNA damage. Despite intensive investigations into the regulation of gene expression in skin cells, the precise molecular events in most cases remain to be elucidated. Novel methods should be investigated in this field.

MicroRNAs (miRNAs) are short, noncoding RNAs of approximately 22 nucleotides that are thought to regulate gene expression through sequence-specific base pairing with the 3′-untranslated region (3′'-UTR) of target mRNAs. To elucidate the molecular mechanisms underlying photodamage, especially skin carcinogenesis by UVB, several research groups have investigated miRNA expression profiles in UVB irradiated cells using miRNA microarrays[4]-[7]. Guo *et al*.[4] investigated the differential expression profiles of miRNAs in NIH3T3 cells in response to UVB irradiation. Pothof *et al*.[5] found that both miRNA expression changes and stress granule formation were most pronounced within the first hours after UVB irradiation, suggesting that miRNA mediated gene regulation occurs earlier than most transcriptional responses. The miRNA response may be related to the DNA damage response and cell proliferation[6]. However, until now there have been no reports on the action mechanisms of photoprotective drugs from the miRNAs aspect.

We previously demonstrated that, compared with untreated mice, pretreatment of skin with baicalin, isolated from the root of the baical skullcap, significantly decreased the number of epidermal DNA photolesions and the amount of cyclobutane pyrimidine dimers (CPDs) after irradiation with 180 mJ/cm^2^ of UVB[8]. Furthermore, topical application of baicalin resulted in a significant decrease in UVB mediated increases in skin edema, skin hyperplasia and leukocyte infiltration. Baicalin treatment also caused a significant decrease in UVB mediated generation of H_2_O_2_ and formation of CPDs[9]. These findings suggest that baicalin protects mouse skin from UVB mediated cutaneous damage. However, the molecular mechanisms underlying this protective effect are largely unknown.

To investigate the protective role of baicalin against UVB irradiation, focusing particularly on the DNA damage repair pathway, we obtained miRNA expression profiles from UVB irradiated mouse skin with or without baicalin treatment using miRNA microarrays.

## MATERIALS AND METHODS

### Animals and UV light source

Animal care and handling were done in compliance with the protocols approved by the Institutional Animal Care and Use Committee of Nanjing Medical University and measures were taken to minimize pain or discomfort. Female C57BL/6 mice (12-week-old) were obtained from the Chinese Academy of Science, Shanghai SLAC Laboratory Animal Co. Ltd (Shanghai, China) and maintained in a specific pathogen-free facility at Nanjing Medical University. The source of UVB was a BLE-1T158 (Spectronics Corp., Westbury, NY, USA). A Kodacel filter (TA401/407; Kodak, Rochester, NY, USA) was used to block the UV of less than 290 nm in wavelengths (UVC). The UVB dose was quantified using a Waldmann UV meter (model no. 585100; Waldmann Co., VS-Schwenningen, Germany) and 180 mJ/cm^2^ of UVB was delivered to the dorsal skin of each mouse.

### Animal treatments

Purified baicalin was purchased from the National Institute for the Control of Pharmaceutical and Biological Products (Beijing, China). All mice were shaved at the beginning of the study. For the detection of CPDs, C57BL/6 mice were allocated into the following four groups. Mice in each group either received no treatment (*n* = 6) or topical baicalin (1 mg/cm^2^ skin area/mouse/100 µL acetone) on their dorsal skin (*n* = 6) or UVB irradiation (180 mJ/cm^2^) (*n* = 12). Six mice were sacrificed 1 and 24 h postirradiation, respectively. In addition, mice received topical baicalin treatment (1 mg/cm^2^ skin area/mouse/100 µL acetone) (*n* = 12), followed by UVB irradiation (180 mJ/cm^2^) 24 h after baicalin treatment, six mice were sacrificed 1 and 24 h postirradiation , respectively.

For the miRNA microarray experiment, C57BL/6 mice were divided into four groups (*n* = 3 per group). The mice either received no treatment or UVB irradiation (180 mJ/cm^2^) or topical baicalin (1 mg baicalin in 100 µL acetone/cm^2^ mouse skin). In addition, mice received topical baicalin (1 mg baicalin in 100 µL acetone/cm^2^ mouse skin), followed by UVB irradiation (180 mJ/cm^2^) 24 h after treatment; 24 h after UVB irradiation, the dorsal irradiated skin was collected for analysis.

### Determination of CPD-positive cells

Skin samples obtained from both the control and treated mice were fixed with 10% formalin and embedded in paraffin. Skin samples were then processed conventionally before paraffin embedding. Serial 6-µm sections were cut, deparaffinized and rehydrated. Endogenous peroxidase activity was blocked by 10 min incubation with 3% hydrogen peroxide; the slides were then incubated with 0.125% trypsin at 37°C for 10 min followed by 1 N HCl at room temperature for 30 min. The slides were washed with PBS and then incubated with 10% goat serum in PBS for 30 min. Sections were incubated with CPD-specific monoclonal antibody (Sigma-Aldrich, St. Louis, MO, USA). Bound anti-CPD antibody was detected by incubation with biotinylated goat anti-mouse IgG1 followed by peroxidase-labeled streptavidin. Sections were then incubated with AEC substrate (Maixin Bioengineering Co., Fuzhou, China) for 5 min. The sections were rinsed with distilled water, and then the slides were observed under a microscope and pictures were captured using a digital camera. All samples were coded and evaluated by two investigators in a blind manner and the mean-SD values were calculated from the evaluation of multiple fields in each group. Five to 10 random fields were counted, and the data represent the results from mice in each group.

### RNA isolation and miRNA microarray

Total RNA isolation and the miRNA enrichment procedure were performed using a mirVana miRNA Isolation Kit (Ambion, Austin, TX, USA) according to the manufacturer's instructions. RNA concentration was quantified with a NanoDrop spectrophotometer (Thermo Fisher, Waltham, MA, USA). The integrity of the RNA was evaluated using an Agilent 2100 Bioanalyzer (Agilent Technologies, Santa Clara, CA, USA). RNA labeling and hybridization on the Agilent miRNA microarray chips were performed using an miRNA Labeling Reagent and Hybridization Kit (Agilent Technologies) at 37°C for 30 min. One hundred nanograms of each total RNA sample was treated with calf intestine alkaline phosphatase (Takara, Dalian, China), denatured using 100% Dimethyl sulfoxide (DMSO, Sigma) at 100°C for 8 min in a thermal cycler and then transferred to an ice-water bath to prevent reannealing of the RNA. The RNA samples were then labeled with pCp-Cy3 using T4 RNA ligase (Ambion) and incubated at 16°C for 2 h. The labeled samples were hybridized to Agilent mouse miRNA microarrays, which contain probes for 627 mouse miRNAs and 39 mouse viral miRNA cataloged in the Sanger Centre Database version 10.1 (http://microrna.sanger.ac.uk). Hybridizations were performed in SureHyb chambers (Agilent Technologies) at 55°C for 24 h. The microarrays were then washed with Agilent prepared buffers. The microarray images were scanned with the Agilent microarray scanner, gridded and analyzed using Agilent Feature Extraction Software version 9.5.1 (Agilent Technologies, Sant Clara, CA, USA). Normalization was performed using the per-chip median normalization method and the median array[10].

### Quantitative real-time PCR analysis for miRNA expression

Expression levels of mmu-miR-233, mmu-miR-141, mmu-miR-23a and mmu-miR-181b were validated using quantitative real-time PCR (qRT-PCR). Primers for qRT-PCR were synthesized by Invitrogen (Shanghai, China) and the primer sequences were as follows:mmu-miR-233, 5′-tgtcagtttgtcaaatacccca-3′,mmu-miR-141, 5′-cataacactgtctggtaaagatgg-3′, mmu-miR-23a, 5′-atcacattgccagggatttc-3′, mmu-miR-181, 5′-aacattcattgctgtcggtg-3′ and mmu-actin, 5′-gatacagagaagatttagcatgg-3′. cDNA synthesis was performed using an miScript Reverse Transcription Kit (Qiagen, Hilden, Germany) according to the manufacturer's protocols. qRT-PCR was performed using an miScript SYBR Green PCR Kit (Qiagen, Hilden, Germany). The reactions were incubated in a 96-well optical plate at 95°C for 15 min, followed by 40 cycles at 94°C for 15 s, at 55°C for 30 s and 70°C for 30 s. Expression analysis was performed in triplicate for each sample. miRNA expression levels were quantified using an ABI Prism 7300 Sequence Detection System (Applied Biosystems, Foster City, CA, USA) with mmu-actin as the normalization control.

### Target prediction and function analysis

TargetScan software was used to predict miRNA targets. To evaluate the TargetScan target predictions for all single miRNAs, we searched for significantly over-represented Gene Ontology (GO) terms among all target genes for all differential miRNAs, respectively, using GOstat software (http://gostat.wehi.edu.au/cgi-bin/goStat.pl)[11]. In brief, the program determines all annotated GO terms and all GO terms that are associated (i.e. in the path) with these for all the genes analyzed. It then counts the number of appearances of each GO term for the genes inside the group and for the reference genes. First, we pasted the mouse RefSeq ID of the target genes into the text area; we then chose “mgi” (*Mus musculus*) from the available GO gene-association databases. For the remaining options, we selected the default values. Often, the most significant GO terms all represent the same subset of genes, because the genes may have several GO annotations that are similar. Fisher's exact test was performed to judge whether the observed difference was significant. For each GO category, this resulted in a *P*-value that the observed counts were due to chance. In addition, pathway analysis of the targets was performed using DAVID Bioinformatics Resources 2008 (http://david.abcc.ncifcrf.gov/).

### Statistical analysis

For statistical analysis, data were analyzed using the SPSS 11.0 (SPSS, Chicago, IL, USA). The statistical significance of difference between groups was determined by one-way analysis of variance (one-way ANOVA) followed by Bonferroni's *t*-test for multiple comparisons. *P* < 0.05 was considered statistically significant.

To identify miRNA that was differentially expressed among groups, Student's *t*-test was performed. Results were considered statistically significant if the *P*-value was less than 0.05.

The threshold cycle (Ct) value for the genes was determined using SDS software version 1.2 (Applied Biosystems, Foster City, CA, USA). The Ct is the cycle number at which fluorescence is generated as a reaction crosses the threshold. The expression levels of mmu-miR-233, mmu-miR-141, mmu-miR-23a and mmu-miR-181b were normalized by subtracting their Ct values from that of the internal control mmu-actin, to obtain ^Δ^Ct. The ^ΔΔ^Ct method for relative quantitation of gene expression was used to determine miRNA expression levels. ^Δ^Ct was calculated by subtracting the Ct of actin from the Ct of the miRNA of interest. ^ΔΔ^Ct was calculated by subtracting the ^Δ^Ct of the reference sample (control) from the ^Δ^Ct of each sample. Fold change was generated using the equation 2^ΔΔ^Ct. All experiments were performed in triplicate. Statistical significance was measured using Student's *t*-test; differences were considered significant at *P* < 0.05.

## RESULTS

### Baicalin attenuates DNA photodamage

**Fig. 1:**
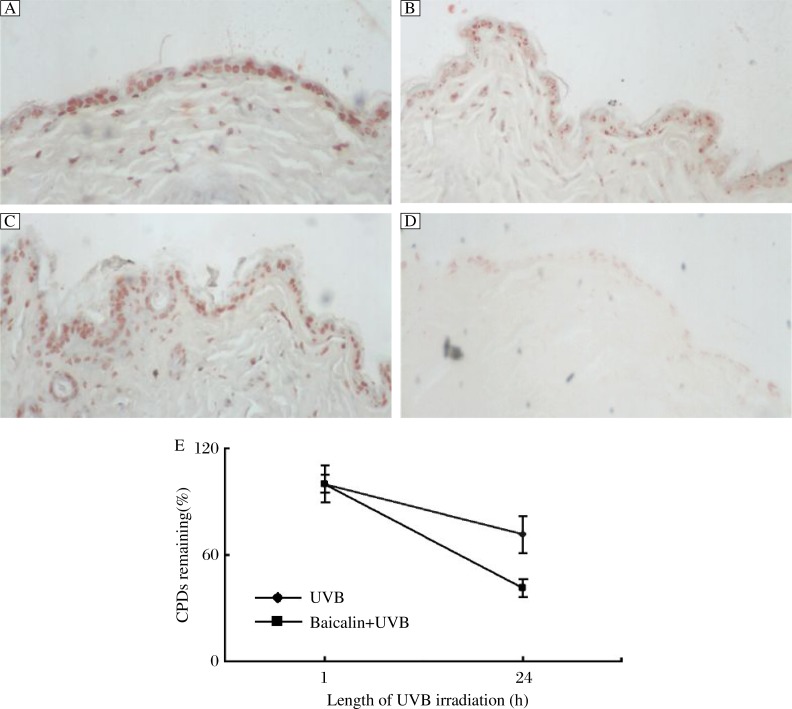
Effects of baicalin on UVB mediated induction of epidermal cyclobutane pyrimidine dimers (CPDs) in mouse skin. From skin biopsies, 6 µm thick sections were cut and the effects of treatments on CPDs were assessed by immunohistochemical analysis. Representative pictures are shown. Details of experiments are provided in the text. A: UVB for 1 h. B: UVB for 24 h. C: Baicalin+UVB for 1 h. D: Baicalin+UVB for 24 h. E. Statistical analysis shows fewer remaining CPDs in the baicalin treated irradiated group than that in the UVB group.

We determined the effect of baicalin on UVB exposure mediated modulations of the CPD markers of DNA photodamage. As expected, UVB exposure resulted in a marked increase in CPDs positive cells in mouse epidermis and upper dermis at 24 h postirradiation. Topical application of baicalin was found to result in a decrease in CPDs at 24 h postirradiation (***Fig. 1***). Statistical analysis also showed that fewer CPDs remained after 24 h in the baicalin-treated irradiated group than that in the UVB irradiated only group (***Fig. 1***).

### Baicalin modulates MiRNA expression in UVB treated mouse skin

We conducted miRNA expression profiling studies of a subset of UVB treated and baicalin plus UVB treated samples using Agilent miRNA microarrays. We identified six miRNAs that were differentially expressed in the control and UVB samples and four miRNAs that were differentially expressed in the UVB and baicalin plus UVB treated samples. Our results suggested these differentially expressed miRNAs may be associated with the mechanisms of photodamage and baicalin photoprotection.

Six miRNAs (mmu-miR-188-5p, mmu-miR-223, mmu-miR-22, mmu-miR-125a-5p, mmu-miR-146a and mmu-miR-141) were found to be differentially expressed in the control group compared with the UVB treatment group (*P* < 0.05, Table S1). Four miRNAs (mmu-miR-23a, mmu-miR-378*, mmu-miR-199a-3p and mmu-miR-181b) were found to be differentially expressed in the UVB group compared with the baicalin plus UVB treated group (*P* < 0.05). The normalized data for differentially expressed miRNAs are shown in supplementary table S1.

**Fig. 2:**
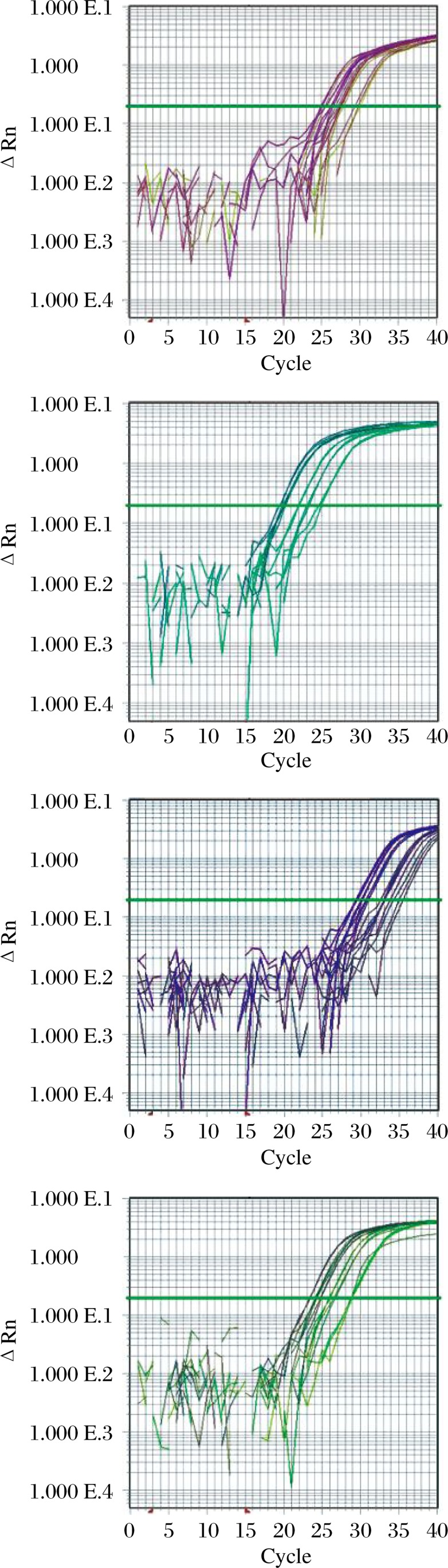
qRT-PCR analysis of miRNA expression. Plots depict the relative change in fluorescence (ΔRn) indicative of product accumulation over 40 cycles of PCR amplification using primer sets specific for the indicated miRNA sequences. PCR reactions were performed in triplicate using cDNA products derived from the indicated groups. In addition, mmu-Actin RNA was similarly analyzed as an internal reference. A: mmu-miR-223. B: mmu-miR-23a. C: mmu-miR-141. D: mmu-miR-181b. E: mmu-Actin.

**Fig. 3:**
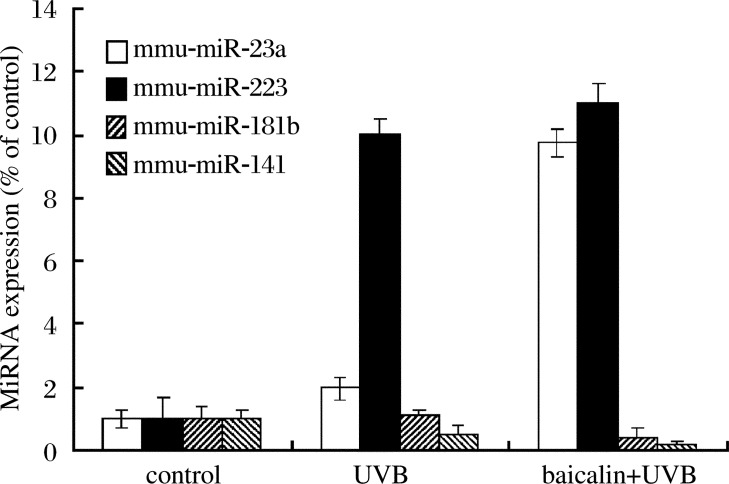
qRT-PCR analysis of selected miRNA expression. The figure shows that mmu-miR-23a is highly expressed in the baicalin plus UVB group compared with the UVB group, and mmu-miR-223 is increased by more than ten fold in UVB treated skin compared with control.

To confirm the microarray findings, we measured the expression levels of four miRNAs (mmu-miR-223, mmu-miR-141, mmu-miR-23a and mmu-miR-181b) that may be related to the regulation of cellular processes using qRT-PCR. The results showed that mmu-miR-23a was highly expressed in the baicalin plus UVB group compared with the UVB group, whereas there was no significant difference between the control group and the UVB group. Expression of mmu-miR-223 was increased by more than ten fold in UVB treated skin compared with the control. mmu-miR-181b was expressed at a lower level in the baicalin plus UVB group, but this was not significantly different from the control. In addition, mmu-miR-141 was down-regulated following UVB irradiation. These results suggest that the expression levels of these four miRNAs observed in the arrays was consistent with those observed using qRT-PCR (***Fig. 2*** and ***Fig. 3***).

### Target prediction and function analysis of differentially expressed miRNA

Prediction of miRNA-regulated gene targets is a necessary step in understanding the functions of miRNA. We used TargetScan to obtain predicted gene targets for all differentially expressed miRNAs. As expected, these miRNA genes could potentially regulate several hundred targets. We then examined the significant GO categories and Kyoto Encyclopedia of Genes and Genomes pathways. Prediction of miRNA-regulated gene targets is a necessary step in understanding the functions of miRNA. We used TargetScan to obtain predicted gene targets for all differentially expressed miRNAs. As expected, these deregulated miRNAs could potentially regulate several hundred targets(***Table 1***). We then examined the significant GO categories and Kyoto Encyclopedia of Genes and Genomes pathways. The deregulated miRNAs may be involved in a diversity of cellular activities including biopolymer metabolic process and regulation of gene expression. (***Tables 2***). And the deregulated miRNAs may contribute to pathways as shown in ***Table 3***.

**Table 1:** Differentially expressed miRNAs and their putative targets

Results	MiRNA	Published targets	Putative targets
Three upregulated miRNAs in the UVB group versus the control group	mmu-miR-188-5p		*RSPO3, PHF3, RNF144A, ZFP91, RAP2C*
	mmu-miR-223	*Mef2c*	*PHF20L1, GPR155, RHOB, FBXW7, NFAT5*
	mmu-miR-22		*FUT9, CLIC4, SNX30, PTGS1, TET2*
Three downregulated miRNAs in the UVB group versus the control group	mmu-miR-125a-5p		*MSRB3, EIF1AD, IRF4, TMEM170B, ENPEP*
	mmu-miR-146a		*IRAKI, TRAF6, APPL1, FAM62B, MED1*
	mmu-miR-141	*ZEB1/TCF8*	*AKAP2, PALM2-AKAP2, ZEB2, FAT3, TRHDE*
One upregulated miRNA in the bcaicalin + UVB group versus the UVB group	mmu-miR-23a	*CXCL12*	*FUT9, ETNK1, RAB39B, KIAA1467, TOPI*
		*FLJ13158*	
Three downregulated miRNAs in the baicalin + UVB group versus UVB group	mmu-miR-378*		*Chmp4c, Tex22, Nme6, Psmd3, Hspa5*
	mmu-miR-199a-3p		*CD 151, CELSR2, CXorf39, FKTN, ACVR2A*
	mmu-miR-181b	*VSNL1, GRIA2*	*KIAA2022, YTHDC2, FIGN, SSX2IP, TRIM2*

**Table 2:** Gene Ontology (GO) terms for the target genes of treatment responsive miRNAs

Results	GO term	Function	*P*-value
Three upregulated miRNAs in the UVB group versus the control group	GO:0043283	Biopolymer metabolic process	3.64E-18
	GO:0050794	Regulation of cellular process	3.64E-18
	GO:0050789	Regulation of biological process	3.56E-17
	GO:0065007	Biological regulation	3.56E-17
	GO:0031323	Regulation of cellular metabolic process	4.37E-15
Three downregulated miRNAs in the UVB group versus the control group	GO:0043283	Biopolymer metabolic process	9.13E-16
	GO:0050789	Regulation of biological process	3.06E-13
	GO:0005515	Protein binding	3.06E-13
	GO:0050794	Regulation of cellular process	3.06E-13
	GO:0010468	Regulation of gene expression	3.06E-13
One upregulated miRNA in the bcaicalin + UVB group versus the UVB group	GO:0043283	Biopolymer metabolic process	6.10E-19
	GO: 0003 700	Transcription factor activity	1.49E-16
	GO:0050794	Regulation of cellular process	1.64E-14
	GO: 0006355	Regulation of transcription, DNA-dependent	1.81E-14
	GO: 0006351	Transcription, DNA-dependent	1.81E-14
Three downregulated miRNAs in the baicalin + UVB group versus UVB group	GO:0010468	Regulation of gene expression	9.13E-16
	GO: 0045449	Regulation of transcription	3.06E-13
	GO: 0006355	Regulation of transcription, DNA-dependent	3.06E-13
	GO:0031323	Regulation of cellular metabolic process	3.06E-13
	GO:0019219	Regulation of nucleobase, nucleoside, nucleotide and nucleic acid metabolic process	3.06E-13

**Table 3:** Pathway analysis of target genes of treatment responsive miRNAs using DAVID Bioinformatics Resources 2008

Results	KEGG pathway	-ln(*P*-value) (Union)
Three upregulated miRNAs in the UVB group versus the control group	ErbB signaling pathway	9.45
	MAPK signaling pathway	8.01
	Dorsoventral axis formation	6.67
	Prostate cancer	6.53
	Chronic myeloid leukemia	5.94
Three downregulated miRNAs in the UVB group versus the control group	Chronic myeloid leukemia	13.54
	Notch signaling pathway	9.32
	Dorsoventral axis formation	7.83
	MAPK signaling pathway	6.29
	Pancreatic cancer	5.67
One upregulated miRNA in the bcaicalin + UVB group versus the UVB group	Renal cell carcinoma	17.12
	Tight junction	14.58
	Focal adhesion	8.74
	MAPK signaling pathway	8.18
	ErbB signaling pathway	7.4
Three downregulated miRNAs in the baicalin + UVB group versus UVB group	Dorsoventral axis formation	18.09
	Glioma	14.46
	Renal cell carcinoma	12.22
	Long term potentiation	10.97
	MAPK signaling pathway	10.92

## DISCUSSION

UVB radiation is the carcinogenic factor in sunlight; damage to skin cells from repeated exposures can lead to the development of cancer[12]. There are cellular mechanisms to repair the DNA damage, or to induce apoptosis to remove severely damaged cells. Our previous findings indicate that baicalin may accelerate CPD removal after UVB irradiation[8]. We have also shown that topical application of baicalin resulted in a significant decrease in acute UVB mediated increases in skin edema, skin hyperplasia and infiltration of leukocytes[9]. However, the underlying mechanism remains unclear. Recent studies have implicated miRNAs in the regulation of proliferation, differentiation and apoptosis; it seems possible that miRNAs might also contribute to the cellular mechanisms of the accelerated DNA damage repair induced by baicalin after UVB irradiation. Our present study reveals that miRNAs are sensitive to UVB and baicalin treatment. We analyzed skin tissues from mice in four groups (those irradiated with UVB, irradiated mice treated with baicalin, mice treated with baicalin only, and a control group) 24 h postirradiation. We found that 3 miRNAs (mmu-miR-125a-5p, mmu-miR-146a, and mmu-miR-141) were downregulated and another three (mmu-miR-188-5p, mmu-miR-223 and mmu-miR-22) were upregulated in UVB irradiated mice compared with untreated mice. Additionally, these miRNAs were predicted to be related to photocarcinogenesis, hypomethylation and apoptosis. Three miRNAs (mmu-miR-378, mmu-miR-199a-3p and mmu-miR-181b) were downregulated and one (mmu-miR-23a) was upregulated in baicalin treated mice compared with UVB irradiated mice, and they were predicted to be related to DNA repair signaling pathway.

Among the UVB deregulated miRNAs, miR-141 has been described as a member of the miR-200 family. Korpal *et al*. [12] found that miR-200 family miRNAs inhibited epithelial-mesenchymal transition and cancer cell migration by direct targeting of the E-cadherin transcriptional repressors ZEB1 and ZEB2. These findings suggest that the downregulated expression of miR-141 induced by UVB irradiation may play a critical role in the repression of E-cadherin, thereby enhancing migration and invasion during cancer progression. The base excision repair protein MED1 is a predicted target of mmu-miR-146a. MED1 interacts with the mismatch repair protein MLH1 and has a central role in the maintenance of genomic stability with dual functions in DNA damage response and repair[14],[15]. MED1 acts as a thymine and uracil DNA *N*-glycosylase on T:G and U:G mismatches that occur at cytosine-phosphate-guanine (CpG) methylation sites due to the spontaneous deamination of 5-methylcytosine and cytosine, respectively. This indicates that MED1 is involved in the removal of methylated DNA[16]. Abnormal DNA methylation (both hypermethylation and hypomethylation) is a hallmark of most cancers, including colon, lung, prostate and breast cancers, and contributes to carcinogenesis by silencing tumor suppressor genes, upregulating oncogenes and/or reducing genomic stability[17],[18]. Mittal *et al*.[19] observed global DNA hypomethylation and reduced maintenance methylation in UV-exposed mouse skin. In view of these findings, whether downregulated expression of miR-146a contributes to UV-induced DNA hypomethylation *via* MED1 deserves further investigation.

In the present study, it is clear that mmu-miR-188-5p, mmu-miR-223 and mmu-miR-22 were upregulated after UVB irradiation. Johnnidis *et al*.[20] found that miR-223 mutant mice have an expanded granulocytic compartment resulting from increases in the number of granulocyte progenitors. They also showed that Mef2c, a transcription factor that promotes myeloid progenitor proliferation, is a target of miR-223[20]-[22]. Their data support a model in which miR-223 acts as a fine-tuner of granulocyte production and inflammatory response. We demonstrated that miR-223 was expressed in untreated mice skin, and markedly increased after UVB irradiation. These findings suggest that mmu-miR-223 is sensitive to UVB and may elicit a direct or indirect effect of UVB-induced inflammation. RhoB, a predicted target of miR-223, has been reported to protect human keratinocytes from UVB-induced apoptosis through epidermal growth factor receptor signaling[23], suggesting that miR-223 may play a role in regulating keratinocyte survival after UVB exposure. However, little is known about the function of miR-188-5p and miR-22.

MiR-23a appeared to be the only upregulated miRNA in baicalin treated and irradiated mice. In a previous study, miR-23a was identified as a growth and localization miRNA in hematopoietic progenitor cells and neuron development[23]. MiR-23a has also been reported to target at the gene encoding stromal cell-derived factor 1 alpha (CXCL12), which is involved in several physiological processes in the skin[24]. CXCL12 interacts with CXCR4 and plays a key role in angiogenesis and the migration of activated Langerhans' cells from the epidermis to the dermis[25]. We predict that changes in miR-23a expression in the skin may stimulate cell differentiation and cell homing. MiR-23a has also been predicted to target the gene encoding topoisomerase I (Top1), which plays an essential role in DNA repair[26]. An increase in the global genomic repair (GGR) of CPDs has been observed with impaired Top1 function in *Saccharomyces cerevisiae*[27]. It is inferred that Top1 in DNA complexes near CPDs may inhibit GGR recognition of these lesions and lead to protein-linked DNA breaks, resulting in CPD repair by an alternative pathway[27]. Top1, as the possible gene target of miRNA-23a, may be associated with accelerated CPD removal of baicalin shown in the present study.

Other potentially important miRNAs downregulated by baicalin with UV irradiation were found in this study (i.e. mmu-miR-181b, mmu-miR-199a-3p, and mmu-miR-378). In chronic lymphocytic leukemia, miR-181b targets Tcl1, a known oncogene, suggesting that this miRNA is a tumor suppressor[28]. However, Zhang *et al*.[29] reported an increased copy number of miR-181b in mammary tumors, and microarray studies showed over expression of this miRNA in cancer tissue samples. These two opposite behaviors of miR-181b, tumorsuppressor and oncogene, are an example of the complexity of miRNA mediated gene regulation and the role that this class of genes plays in carcinogenesis. From the miRNA miRBase database, (http://microrna.sanger.ac.uk/), may be a target of miR-181b and miR-378*, and plays an important role in the NER signaling pathway. We speculate that downregulation of miR-181b and miR-378* by baicalin may upregulate CPD removal *via* the NER signaling pathway. Although there is no evidence that the *Xpa* gene is the direct target of miR-181b and miR-378*, the prediction of their target genes provides clues for further study. There are no reports associating miR-199a-3p with DNA repair and carcinogenesis. Unfortunately, we did not obtain useful data related to the DNA repair activity of baicalin.

To further analyze the relationship between patterns of target gene expression and their functional implications, we classified the target genes of all differentially expressed miRNAs into several function categories using GOstat software (http://gostat.wehi.edu.au/cgi-bin/goStat.pl)[11]. The targets of these nine miRNAs were chosen for pathway analysis using DAVID Bioinformatics Resources 2008 (http://david.abcc.ncifcrf.gov/).

It should be noted that some of the miRNAs reported to be involved in the response to UVB irradiation were not observed in this study. For example, miR-21 is known to be involved in the progression of cancer and has been described as an oncogenic miRNA[30], but when it appears together with miR-24, it inhibits growth[4]. Guo *et al*.[4] found that miR-21 and miR-24 appeared together at 12 h after exposure to 50 J/m2 UVB and a sub-G1 DNA content fraction and apoptotic cells appeared at 12 h post-irradiation, suggesting that miR-21 and miR-24 together inhibited growth. However, the present study did not find any changes in miR-21 or miR-24 in the UVB group. This may be due to differences in animal selection, UVB dose or chip fabrication or even a weakness of the microarray technology.

In summary, the focus of this study was to investigate the differential expression profiles of miRNAs in mouse skin in response to treatment with baicalin after UVB irradiation. Although this is a preliminary investigation, we believe our study provides a basis for further investigation of the signal transduction pathways induced by UVB and baicalin treatment.
